# Submarine Basaltic Glass Colonization by the Heterotrophic Fe(II)-Oxidizing and Siderophore-Producing Deep-Sea Bacterium *Pseudomonas stutzeri* VS-10: The Potential Role of Basalt in Enhancing Growth

**DOI:** 10.3389/fmicb.2017.00363

**Published:** 2017-03-10

**Authors:** Lisa A. Sudek, Greg Wanger, Alexis S. Templeton, Hubert Staudigel, Bradley M. Tebo

**Affiliations:** ^1^Marine Biology Research Division, Scripps Institution of Oceanography, University of California, San Diego, La JollaCA, USA; ^2^Jet Propulsion Laboratory, California Institute of Technology, University of Southern California, PasadenaCA, USA; ^3^Department of Geological Sciences, University of Colorado Boulder, BoulderCO, USA; ^4^Institute of Geophysics and Planetary Physics, Scripps Institution of Oceanography, University of California, San Diego, La JollaCA, USA

**Keywords:** biofilm, basalt, Fe(II) oxidation, microbial fuel cell, siderophores, diffusion chamber

## Abstract

Phylogenetically and metabolically diverse bacterial communities have been found in association with submarine basaltic glass surfaces. The driving forces behind basalt colonization are for the most part unknown. It remains ambiguous if basalt provides ecological advantages beyond representing a substrate for surface colonization, such as supplying nutrients and/or energy. *Pseudomonas stutzeri* VS-10, a metabolically versatile bacterium isolated from Vailulu’u Seamount, was used as a model organism to investigate the physiological responses observed when biofilms are established on basaltic glasses. In Fe-limited heterotrophic media, *P. stutzeri* VS-10 exhibited elevated growth in the presence of basaltic glass. Diffusion chamber experiments demonstrated that physical attachment or contact of soluble metabolites such as siderophores with the basaltic glass plays a pivotal role in this process. Electrochemical data indicated that *P. stutzeri* VS-10 is able to use solid substrates (electrodes) as terminal electron donors and acceptors. Siderophore production and heterotrophic Fe(II) oxidation are discussed as potential mechanisms enhancing growth of *P. stutzeri* VS-10 on glass surfaces. In correlation with that we discuss the possibility that metabolic versatility could represent a common and beneficial physiological trait in marine microbial communities being subject to oligotrophic and rapidly changing deep-sea conditions.

## Introduction

Microorganisms play a pivotal role in the geochemical cycling of elements and the overall ecological function of deep-sea aquatic systems (Deming and Baross, 1993; Edwards et al., 2005). In hydrothermally influenced deep-sea habitats, microbes have recently been shown to contribute a substantial fraction to the biogeochemical cycling of Fe, an essential element for almost all living organisms (Li et al., 2013). As observed in surface waters, the bioavailability of Fe controls patterns of primary productivity and carbon cycling in the deep-sea and throughout the oceans (Moore et al., 2002; Boyd and Ellwood, 2010). While hydrothermally active deep-sea systems such as submarine volcanoes and mid-ocean ridge environments are significant sources of fluids enriched in Fe, aqueous Fe(II) rapidly oxidizes and precipitates as Fe(III) oxyhydroxides when mixed with deep, oxygenated seawater. Significant Fe resolubilization can then occur through microbial activity, such as through the chelation of Fe(III) by organic ligands. This “iron pump” was recently identified as an important mechanism for making Fe bioavailable both locally and globally (Statham et al., 2005; Bennett et al., 2008; Toner et al., 2009; Li et al., 2013).

Despite the rapid abiotic oxidation of Fe(II) and the low energetic yield compared to more favorable chemolithotrophic metabolisms such as sulfide oxidation (Edwards et al., 2005; Emerson et al., 2010), both autotrophic and heterotrophic Fe(II)-oxidizing bacteria (FeOB) have been shown to be part of phylogenetically and metabolically diverse deep-sea microbial communities (Edwards et al., 2003; Emerson et al., 2007; Sudek et al., 2009; Singer et al., 2013; Scott et al., 2015). They are thought to play an important role in redox transformations of Fe in deep-sea systems (Edwards et al., 2004), and they are commonly associated with diffuse seeps of Fe(II)-rich fluids (Emerson and Moyer, 2002; Edwards et al., 2011; Scott et al., 2015), as well as basaltic surfaces of varying age (Santelli et al., 2008; Sudek et al., 2009; Henri et al., 2016).

Basaltic glass comprising about 20% of the extrusive layer of the oceanic crust represents one of the most geochemically reactive sources of solid-phase Fe for microbial colonization in the deep-sea and in addition, contains a number of bio-essential elements including Mg, Ca, Mn, and P (Staudigel and Hart, 1983; Drechsel and Winkelmann, 1997). Recent phylogenetic studies have shown that deep-sea basalt surfaces harbor phylogenetically diverse microbial communities (Thorseth et al., 2001; Lysnes et al., 2004; Templeton et al., 2005; Mason et al., 2007, 2009; Santelli et al., 2008; Sudek et al., 2009). Quantitative polymerase chain reaction (qPCR) measurements of microbial communities associated with the glassy rims of young weathered basalts from the East Pacific Rise (EPR) have indicated total bacterial and archaeal cell densities between 3 × 10^6^ and 1 × 10^9^ cells per gram of rock (Santelli et al., 2008). In contrast, the total microbial cell numbers of deep ocean waters (>1000 m) range between 8 × 10^3^ and 9 × 10^4^ cells per ml of seawater (Santelli et al., 2008).

Bacterial metabolisms within such basalt surface communities have been shown to be versatile (Templeton et al., 2005; Mason et al., 2009, 2010; Sudek et al., 2009; Callac et al., 2013). Reduced forms of elements such as Fe(II), Mn(II) and S(-II) in basalts can serve as potential electron donors for chemolithotrophic communities (Christie et al., 2001; Bach and Edwards, 2003; Henri et al., 2016). In addition, chemolithoautotrophic Fe(II)-oxidizing organisms are widely assumed to fix carbon that at least part of the basalt community relies upon (Santelli, 2007).

Metal oxides often found on the basalt surface and formed through the abiotic and biotic oxidation of basalt can also serve as nutritional source of Fe for siderophore-producing bacteria. In fact, many of the microbial strains found in association with submarine basaltic glass surfaces have been shown to be capable of multiple metabolic functions (Sudek et al., 2009). It is unclear to what degree this metabolic plasticity may represent a niche adaptation response or what the advantages of basalt surface-associated growth may offer these communities. In particular it remains ambiguous whether the colonization process is directed toward fulfilling specific ecological advantages such as nutrient acquisition and/or energy generation. Little is known about the microbial ecology of glass colonization, including the key mechanisms that drive surface colonization. Finally it is unclear to what degree microbial activity can affect the dissolution rates of basaltic glasses and hence control the biogeochemical cycling of elements at the rock/water interface. Weathering structures on the surface of basaltic glass surfaces and the occurrence of palagonite have suggested a role of bacteria in the development of the structures (Thorseth et al., 1992, 1995). More recently laboratory batch experiments on basalt samples from near Axial Seamount indicated that basalt weathering (i.e., release of metals such as Fe, Mn and Ca) was increased in the presence of bacteria (Daughney et al., 2004). It was also shown that surface features and mineralogies of basalts weathered in the presence of bacteria were similar to those of natural weathered basalts (Daughney et al., 2004).

Here we investigate the role of submarine basaltic glass in the growth of the siderophore-producing and Fe(II)-oxidizing deep-sea bacterium *Pseudomonas stutzeri* VS-10. This Gammaproteobacterium was isolated from a submarine volcanic rock at Vailulu’u Seamount (Sudek et al., 2009) and, due to exhibiting increased growth in the presence of basalt, was used as a model for studying the characteristics of microbe/basalt interactions. Specifically, we explore the role of basalt as a source for either nutrients or energy and the importance of direct physical contact of the cells or soluble compounds produced by the bacterium with the basalt. In addition, we discuss the potential role of filamentous structures within *P. stutzeri* VS-10 biofilms in surface-related growth. This work provides new evidence for possible roles of submarine basaltic glass in supporting metabolically diverse deep-sea microbial communities.

## Materials and Methods

### Volcanic Rocks and Minerals

The basaltic glass used in all experimental set-ups was originally collected at Kilauea volcano (Pu’U-o’O), Hawaii, USA in 2002 and had been re-melted as described elsewhere (Bailey et al., 2009). Fe(II) content in the basalt averaged 9–10 wt %. For all growth experiments, the basalt was crushed to an approximate grain size of 2–3 mm, washed several times in deionized water, washed twice in 0.1N HCl to remove potential surface Fe, and sonicated in deionized water for 40 min. After a final rinse with deionized water the glass grains were dried in sterile glass dishes, covered with aseptic aluminum foil at 40°C and then microwave sterilized in glass dishes for 10 min.

### Organisms and Media

*Pseudomonas stutzeri* VS-10 was isolated from a rhyolitic rock surface exposed for 3 months to low-temperature, non-hydrothermally influenced conditions (Marker 2: 14 13.103°S / 169 04.106°W; 582 m water depth) at Vailulu’u Seamount. It was isolated as a siderophore producer through serial transfer of colonies on Chrome-Azurol-Sulfonate plates (Sudek et al., 2009) and was shown to grow well at room temperature. All experiments were subsequently carried out at room temperature to ensure reproducibility of results from different experimental set-ups. *Shewanella oneidensis* MR-1 was obtained from K. Nealson at the J. Craig Venter Institute where the electrochemical work was carried out.

#### Minimal Glycerol Medium

An artificial seawater medium amended with glycerol was used in Fe-limited growth experiments in the presence and absence of basalt. It was also used in diffusion chamber experiments to assess the cell’s requirement for basalt as a nutrient source and microbial fuel cell (MFC) experiments to analyze *P. stutzeri* VS-10’s ability to use electrodes as an electron acceptor. The medium contained: 100 mM MgSO_4_, 20 mM CaCl_2_, 600 mM NaCl, 20 mM KCl, 40 mM glycerol, 333 μM K_2_HPO_4_, 18.5 mM NH_4_Cl and 50 mM HEPES (pH 7.4). To limit the potential contamination of the medium with Fe the medium (adjusted to pH 7.4) was passed through a column containing Chelex-100 resin (analytical grade 100–200 mesh sodium form, Bio-Rad cat. # 142-2832) prepared based on the methods of Price et al. (1989). The Chelex was loaded in a previously acid washed (10 % HCl, Fisher cat. # A144-212) 1.5 × 15 cm glass column (Kontes class company flex column, cat. # 420400-1515) equipped with a three-way nylon stopcock (Kontes cat. # 420 163-4503). The flow rate was adjusted to approximately one drop per second. The medium was subsequently filter-sterilized through 0.22 μm Stericups (Millipore cat. #: SCGPU05RELC) and the final pH was measured. To test for the role of basalt as a source for nutrients and energy no trace elements were added.

#### MR-1 Medium for Fuel Cell Experiments

For our MFC experiments we used two different media. Experiments testing for the reduction potential of *P. stutzeri* VS-10 were run on minimal glycerol medium (see Minimal Glycerol Medium With and Without Basalt) to allow for comparison of results to data from growth experiments. In contrast, experiments testing for the oxidation potential of strain VS-10 in combination with *S. oneidensis* MR-1 contained MR-1 defined medium that allowed for comparison of results to previously published data collected on *S. oneidensis* MR-1 (Bretschger et al., 2007). It contained 30 mM PIPES [piperazine-*N*,*N*^prime^-bis(2-ethanesulfonic acid)] pH 7.2, 7.5 mM NaOH, 10 mM NH_4_Cl, 1.34 mM KCl, 4.35 mM NaH_2_PO_4_, 30 mM NaCl with the addition of 10 ml/liter each of vitamin solution (Kieft et al., 1999), amino acid solution and trace mineral stock solution (Bretschger et al., 2007). A final concentration of 20 mM lactate was used as the sole carbon source.

### Growth Experiments

#### Minimal Glycerol Medium With and Without Basalt

Prior to the inoculation of all minimal medium cultures with *P. stutzeri* VS-10, the strain was grown at 37°C overnight in Luria-Bertani (LB) medium (per L of ultrapure H_2_O: 10 g Bacto Tryptone, 5 g Yeast Extract, 10 g NaCl, adjusted to pH 7.0). Cells were harvested by centrifugation at 8000 rpm for 10 min. and washed three times in 5 ml of minimal glycerol medium. The optical density at 600 nm (OD_600_) of the final resuspension of cells was measured to estimate cell numbers. Fifteen ml glass tubes, previously washed in 10% HCl for 2 days, rinsed six times in deionized water and microwave-sterilized for 10 min., were used to determine growth of *P. stutzeri* VS-10 on minimal glycerol medium in the presence and absence of basalt. In the tubes containing basalt ∼0.5 g of basaltic rock grains were added to 5 ml of Chelexed medium. Except for abiotic (rock/water) controls each tube was subsequently inoculated with the washed cell suspension of *P. stutzeri* VS-10.

For the removal of biofilms from the interior of the glass tubes and the rock and mineral grains prior to spectrophotometric cell density measurements at 600 nm, the tubes were briefly vortexed and vigorously tapped onto a surface. Scanning electron microscopy (SEM) later showed the effectiveness of this method in removing bacterial cells from the rock surfaces. Due to the size and density of rock and mineral grains and their rapid settling after vortexing, they are unlikely to have interfered with the optical density measurements. To account for any contribution from abiotically derived alteration products, all cultures were compared with controls (rock/water, mineral/water), which represented less than 2% of the measured OD of the cultures. The interference of biologically derived secondary mineral deposits as well as small amounts of Fe hydroxides inside the culture containing FeCl_2_ with OD_600_ measurements was considered insignificant.

#### Diffusion Chamber

The significance of direct attachment or proximity of cells or soluble compounds produced by the bacterium to the basalt surface for growth of *P. stutzeri* VS-10 was investigated in diffusion chambers, consisting of dual compartment borosilicate chambers similar to the one described in Bretschger et al. (2007). We use the term “proximity” in this manuscript to refer to the absence of a physical barrier between the cells and the basalt where soluble molecules such as siderophores or redox-active compounds could come in contact with the basalt surface. The diffusion chamber compartments were separated by a proton-exchange membrane (Nafion^®^ 424, DuPont) which would be impermeable to small and large molecules and colloids but did not contain any electrodes. Prior to inoculation with *P. stutzeri* VS-10 cells, 5 g of sterile basalt was added to one of the compartments. The glass chambers were assembled, wrapped in aluminum foil and autoclaved at 121°C for 30 min. Upon cooling, 50 ml of filter sterilized minimal glycerol medium was added to both compartments. Nutrient-limited washed cells (see above) of *P. stutzeri* VS-10 were added to an initial OD_600_ of ∼0.05 to the compartment containing basalt grains in one set-up and to the opposite side in the other set-up. Cell growth was subsequently monitored based on optical density measurement at 600 nm. The abiotic status of the uninoculated compartment was continuously monitored based on optical density measurements and microscopic analysis. A constant flow of filter-sterilized air was applied to both sides of each chamber to assure sufficient oxygen concentrations for the growth of the strain and to support homogenization of the solution prior to optical density measurements. Biofilm formation on the inside walls of the glass chamber was minimized by vigorous shaking and careful tapping, resulting in the visible detachment and homogenization of biofilms from the glass walls. Using sterile pipets the solution was again homogenized prior to sampling.

### Scanning Electron Microscopy

Scanning electron microscopy was utilized to investigate biofilm formation and morphology of *P. stutzeri* VS-10 on basaltic glass surfaces. To preserve the cell integrity of all samples they were initially fixed in 2.5% glutaraldehyde (v/v) in 0.1 M sodium cacodylate buffer (pH 7, Fisher cat. # NC9842332) at 4°C overnight. To remove salts the samples were washed with 30 mM followed by 15 mM 2-[4-(2-sulfoethyl)piperazin-1-yl]dethanesulfonic acid (PIPES, pH 7.4). The samples were then gradually dehydrated by washing with a graded series of 10, 25, 50, 75, 90, and finally 100% ethanol for 5 min each. To prevent deformation and collapse of the surface structure and to conserve surface morphology samples were critical point dried. After mounting the samples on SEM stubs they were sputter-coated with gold/palladium (80/20) in a coating unit at V = 2.5 KV and I = 20 mA for 60 s. Samples were imaged on a Philips XL30 field emission SEM (FESEM) at the Nano3 facility of the University of California, San Diego.

### Microbial Fuel Cells

Electrochemical properties of the *P. stutzeri* VS-10 biofilm under nutrient-limited conditions were investigated in MFCs. Current production was observed using dual chamber MFCs of the type shown in Bretschger et al. (2007). MFCs were assembled using proton-exchange membranes (Nafion^®^ 424, DuPont) and electrodes. In MFC experiments testing for *P. stutzeri* VS-10’s ability to use a cathode as a terminal electron donor (oxidation potential) both anode and cathode were constructed from graphite felt (GF-S6-06, Electrolytica). In experiments testing for the strain’s reduction potential the anode was constructed from graphite felt (GF-S6-06, Electrolytica) while the cathode was graphite bonded to platinum wire (0.3 mm, Alfa-Aesar). The cathode electrodes in both experiments were electroplated with a platinum catalyst to drive the oxygen reduction reaction. The MFCs were autoclaved prior to the addition of 0.22 μm filtered media. Ag/AgCl reference electrodes were aseptically inserted into both the anode and cathode compartments after autoclaving. Anaerobic conditions were maintained on the MFC anode by continuously passing filtered nitrogen gas through the compartment at a rate of 20 mL/min. Aerobic conditions were maintained in the cathode compartment by continuously passing air at a rate of 40 mL/min (Manohar et al., 2008).

#### Oxidation Potential

The potential of *S. oneidensis* MR-1 and *P. stutzeri* VS-10 to use a cathode as an electron donor was tested. Three separate sets of MFCs were set up, one abiotic and two biotic. Prior to the experimental set-up the growth of *P. stutzeri* VS-10 on this medium was ensured. Each MFC contained 30 ml of sterile MR-1 defined medium containing 10 mM of lactate as the sole electron donor and carbon source. The anode compartments of these fuel cells were initially inoculated with *S. oneidensis* MR-1 at a density of OD_600_ of 0.6. After 100 h in which the cathode compartments of both MFCs had remained abiotic, the cathode compartments of each MFC was inoculated with, in one case, a culture of *S. oneidensis* MR-1, in the other case, a culture of *P. stutzeri* VS-10. MR-1 consumes the lactate and transfers the electrons to the anode which functions as the electron acceptor. The electrons are then passed on to the cathode where they serve as an electron source for either MR-1 or VS-10 in the cathode compartment. Both strains were grown on MR-1 defined medium, washed as described before (see above) and inoculated to an initial OD_600_ of ∼0.6 (**Table 1**).

**Table 1:** Experimental set-up of microbial fuel cells (MFCs) testing for the oxidation potential of *P. stutzeri* VS-10.

	Anode	Cathode
MFC 1	Abiotic	Abiotic
MFC 2	*S. oneidensis MR-1*	*S. oneidensis MR-1*
MFC 3	*S. oneidensis MR-1*	*P. stutzeri VS-10*

#### Reduction Potential

The ability of *P. stutzeri* VS-10 to reduce an anode, and hence use a solid surface as a terminal electron acceptor, was determined. Thirty milliliters of 0.22 μm filter-sterilized minimal glycerol medium was added into each of the compartments of two MFCs. One served as an abiotic control while the other one was subsequently inoculated with a washed culture of *P. stutzeri* VS-10. Prior to inoculation of the anode compartment, *P. stutzeri* VS-10 was grown to exponential phase on LB medium, washed as described previously (see Growth Experiments) and inoculated to a final OD_600_ of ∼0.05. Throughout the experiment samples were taken through sterilized disposable borosilicate glass Pasteur pipettes (Fisher cat # 13-678-20C) and the OD_600_ of the culture was determined spectrophotometrically. Initially, aerobic conditions inside the cell were established by supplying filter-sterilized oxygen (0.22 μm) to both compartments close to the electrodes. After 73 h conditions were changed from aerobic to anaerobic conditions by replacing the oxygen flow to the anode compartment with filter-sterilized (0.22 μm) nitrogen.

#### Electrochemical Measurements

Microbial fuel cell voltage was measured every minute across a 100 Ω external resistor using a high-impedence digital multimeter (model 2700; Keithley Instruments). MFC polarization measurements were taken for both the oxidation (see Oxidation Potential) and reduction (see Reduction Potential) potentials by opening the circuit and allowing the cell potential to build to a maximum (i.e., Open Circuit Potential or OCP). Once the OCP was reached, a potentiostat (Gamry Inc) was used to measure the polarization behavior of the MFC. During this process the external resistance is decreased from a maximum (infinite resistance) to a minimum (short circuit) and the corresponding current is measured. Current (I) is calculated from these data using Ohm’s law (I = V/R) and Power (P) is calculated using P = I^∗^V. The total surface area of the anode was 290 cm^2^.

The MFCs were also evaluated in the experiment measuring the reduction potential using cyclic voltammetry (CV) to determine the redox activity of the abiotic components (e.g., electrodes) as well as the biotic components (e.g., bacteria). The voltammograms were obtained by sweeping the cell potential and measuring the corresponding current response. The voltage of the electrodes was swept at a scan rate of 25 mV/s from -500 mV to +500 mV vs. Ag/AgCl. At the termination of the experiments the electrodes were harvested, fixed in 2.5% glutaraldehyde (v/v) in 0.1 M sodium cacodylate buffer (pH 7, Fisher cat. # NC9842332) at 4°C overnight and visualized by SEM.

## Results

**FIGURE 1:**
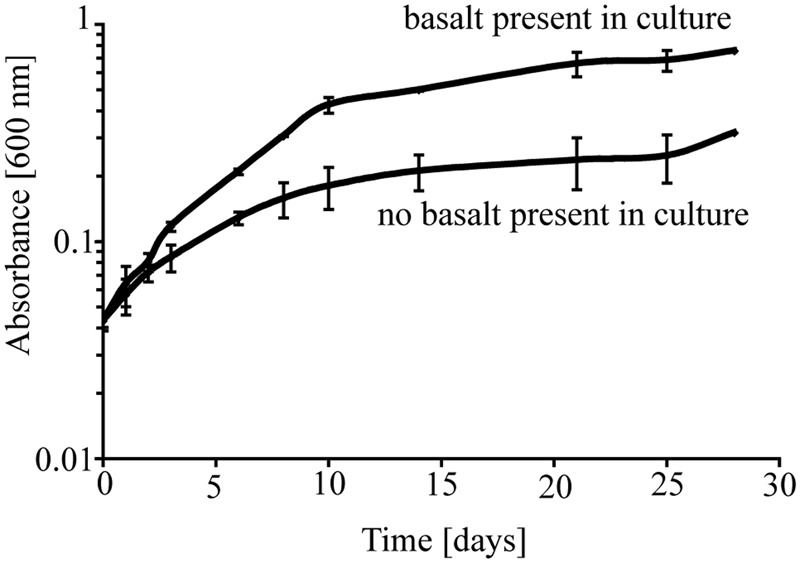
**Growth of *P. stutzeri* VS-10 on Chelex-treated minimal glycerol medium with or without basalt.** Results are based on duplicate growth experiments for each condition. Error bars represent the standard deviation.

To investigate the potential role of basalt in supporting growth of *P. stutzeri* VS-10, we initially carried out two batch growth experiments at room temperature under aerobic Fe-limited conditions using a minimal medium containing glycerol as the sole carbon source, with and without basalt. In the batch experiments occurring over a period of ∼4 weeks, we observed various growth of *P. stutzeri* as measured spectroscopically by light absorption at 600 nm (**Figure 1**). The results showed that while there was some growth (increase from OD_600 nm_ of 0.046–0.316) on the glycerol medium alone, growth in the presence of glycerol and basalt was 2.4 times higher (from 0.045 to 0.754) after 28 days (**Figure 1**).

**FIGURE 2:**
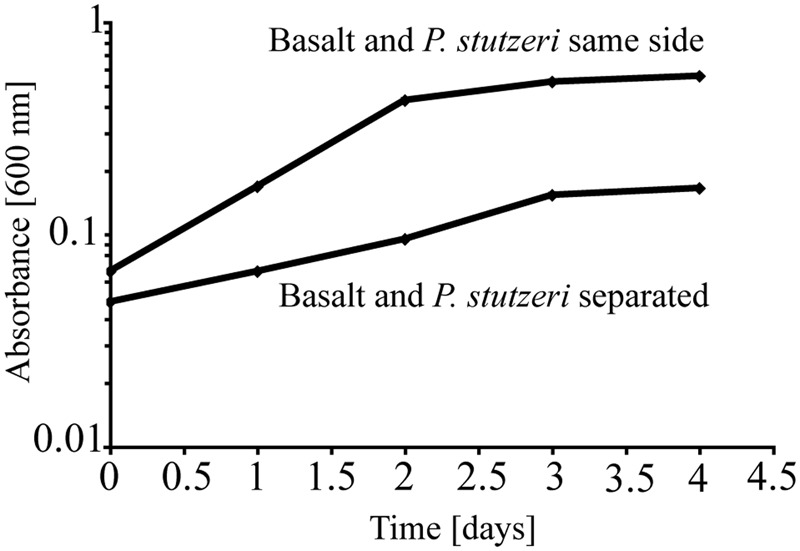
**Diffusion chamber experiments demonstrating growth of *P. stutzeri* VS-10 on Chelex-treated minimal glycerol medium in direct contact with and physically separated from basalt**.

To test the importance of direct attachment or proximity (i.e., lack of a physical barrier) of cells to basaltic glass in contributing to the elevated growth of *P. stutzeri* VS-10, additional growth experiments were conducted in a diffusion chamber. *P. stutzeri* VS-10 was grown under Fe-limited conditions either in the presence of basaltic glass or physically separated from basaltic glass by a membrane. Under physical separation, growth of the bacterium is reduced (**Figure 2**), similar to the results of our first experiment when basalt was absent (**Figure 1**). In contrast, the presence of basalt leads to elevated growth, exceeding the growth observed in our batch experiment, a result likely due to larger amounts of basalt used in the diffusion chamber as well as more constant agitation. These results demonstrate that physical contact, or at least proximity of *P. stutzeri VS-10* to basaltic glass, increases growth.

### Morphological Appearance of *P. stutzeri* VS-10 Biofilms on Basaltic Glass Surfaces

**FIGURE 3:**
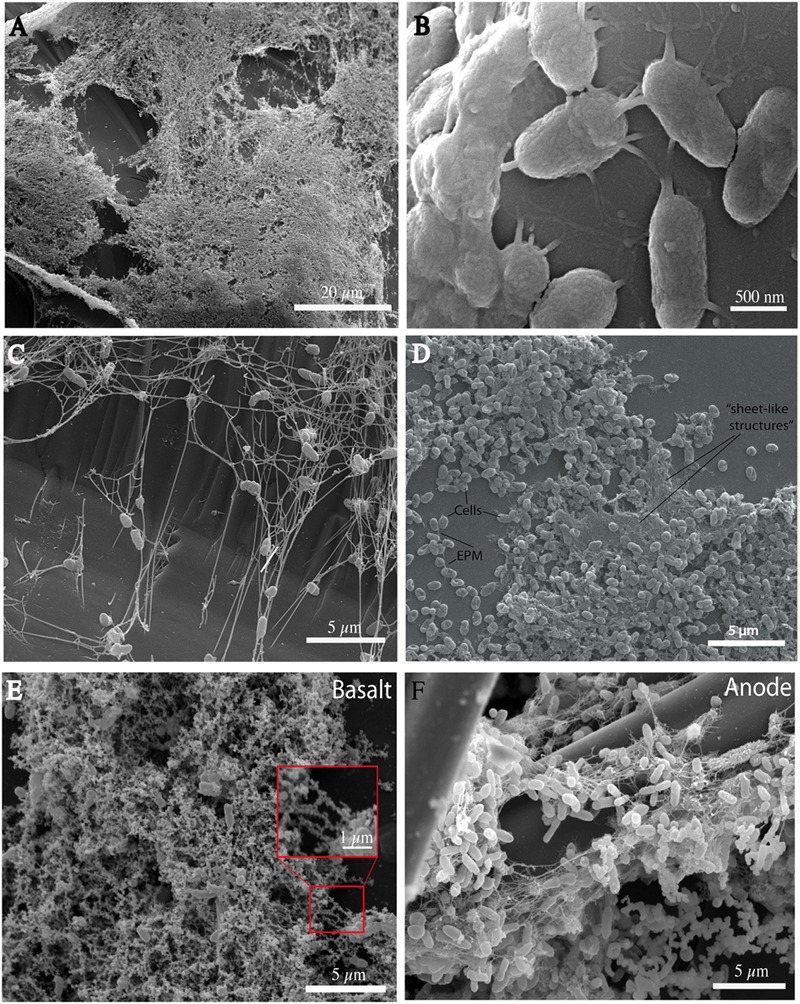
**Scanning electron micrographs (SEMs) of *P. stutzeri* VS-10 cells on basaltic glass taken at different time points.** All cultures were grown on minimal glycerol medium. Basalt fragments used in the cultures (all but **F**) were all treated equally. Biofilm formation is extensive and not limited to natural depressions (**A**, 22 days of incubation). Filamentous structures may facilitate cell-to-cell and cell-to substrate adherence (**B,C**, 4 days of incubation). In thicker parts of the biofilm they appear almost “sheet-like” (**D**, 1 week of incubation). Encrustation of the filaments is apparent when grown in the presence of basalt (red box **E**, after 6 days of incubation). Encrustations appear significantly reduced in biofilms developed inside microbial fuel cells (**F**, anode biofilm).

Having demonstrated that physical contact and/or the exchange of soluble molecules between the bacterium and the glass has an important effect on growth we explored the morphological appearance of *P. stutzeri* in its biofilms ranging from monolayers of cells to more complex structures (**Figure 3**). The biofilm distribution appears to be extensive and not limited to natural depressions (e.g., cracks) in the rock surface (**Figure 3A**). Filamentous structures (with an approximate diameter of 10 nm) are a noticeable part of the VS-10 biofilm (**Figures 3B,C**). These filaments are similar in appearance to fiber-like structures recently described as extensions of the outer membrane in *Shewanella oneidensis* MR-1 (Pirbadian et al., 2014). Structures similar in appearance have also previously been described as artifacts stemming from the dehydration of EPS as part of the SEM preparation (Dohnalkova et al., 2011). Without a detailed compositional analysis a classification of these filaments is impossible.

The role of extensions in *P. stutzeri* VS-10 biofilms appears to be to facilitate both cell-to-cell and cell-to-glass interactions (**Figures 3B,C**). They vary in number ranging from 1 or 2 to up to ∼20 filaments per cell and are unevenly spread over the entire cell surface (**Figure 3B**). The number of appendages on each cell appears to depend on biofilm thickness. Single cells seem to adhere to the glass surface via a few (2–10) appendages (**Figure 3B**). Cells within thicker parts of the biofilm exhibit more and longer extensions or “sheet-like” structures (**Figure 3D**).

When grown on minimal glycerol medium in the presence of basalt the filaments and cells become mineral-encrusted (**Figure 3E**). Energy Dispersive Spectroscopy (EDS) identified iron and oxygen as the main elements present in the encrustations and traces of silica and titanium (data not shown). These analyses suggest that these encrustations likely represent secondary minerals [e.g., Fe(hydr)oxides, Fe(II)/(III)-silicate/oxides and possibly Fe(II)-bearing minerals] formed as a result of basalt weathering.

### Electrochemical Properties of *P. stutzeri* VS-10

To further investigate the role of the biofilm in the elevated growth of *P. stutzeri* VS-10 on basalt, the electrochemical properties of the strain were investigated in MFCs. The oxidation/reduction potential of *P. stutzeri* VS-10 was measured and compared to data obtained from *S. oneidensis* MR-1, a known electrogenic bacterium (El-Naggar et al., 2010).

#### Oxidation Potential

In addition to a blank MFC (MFC1) the anode compartments of two MFCs (MFC2 and 3) were initially inoculated with cultures of *S. oneidensis* MR-1, a bacterium known to couple the oxidation of organic compounds to the reduction of an anode inside MFCs (Fitzgerald et al., 2012). At t_72 h_, when the cathode compartments were still abiotic, the circuit was opened and the potential of the fuel cell was allowed to build to a maximum open circuit potential (OCP). The initial OCP of both MFCs was similar, MFC2: +285 mV and MFC3: +270 mV vs. Ag/AgCl. At t_100 h_ the cathode compartments of MFC2 and MFC3 were inoculated with *S. oneidensis* MR-1 and *P. stutzeri* VS-10, respectively. At t_140 h_ the OCP of MFC2 (with MR-1) and MFC3 (with VS-10) had increased to +344 mV and +398 mV vs. Ag/AgCl respectively (**Table 2**).

**Table 2:** Results from experiments testing for the oxidation potential of VS-10 in Microbial Fuel Cells (MFCs).

	MFC1	MFC2		MFC3	
Cathode/anode	Abiotic/abiotic	MR-1/abiotic	MR-1/MR-1	MR-1/abiotic	MR-1/VS-10
		T_72 h_	T_140 h_	T_72 h_	T_140 h_
Open circuit potential [mV]	–	+270	+344	+285	+398
Maximum current density [μA/m^2^]	–	7.45	23.77	6.49	20.77

*While MFC1 was run as an abiotic blank, both anode compartments of MFC2 and 3 were initially inoculated with a culture of *S. oneidensis* MR-1 while the cathode compartments remained sterile. At 100 h the cathode compartment of MFC2 was inoculated with another culture of *S. oneidensis* MR-1 while the cathode compartment of MFC3 was inoculated with a culture of *P. stutzeri* VS-10.*

The maximum current densities for both MFCs were also recorded before (t_72 h_) and after (t_140 h_) the inoculation of the cathode compartments with the respective strains. Maximum current densities of MFC2 containing *S. oneidensis* MR-1 on the cathode side increased from 7.45 μA/m^2^ at t_72 h_ to 23.77 μA/m^2^ at t_140 h_. Comparatively, the maximum current density in MFC3 containing *P. stutzeri* VS-10 on the cathode side increased from 6.49 μA/m^2^ (t_72 h_) to 20.77 μA/m^2^ (t_140 h_) (**Table 2**).

#### Reduction Potential

**FIGURE 4:**
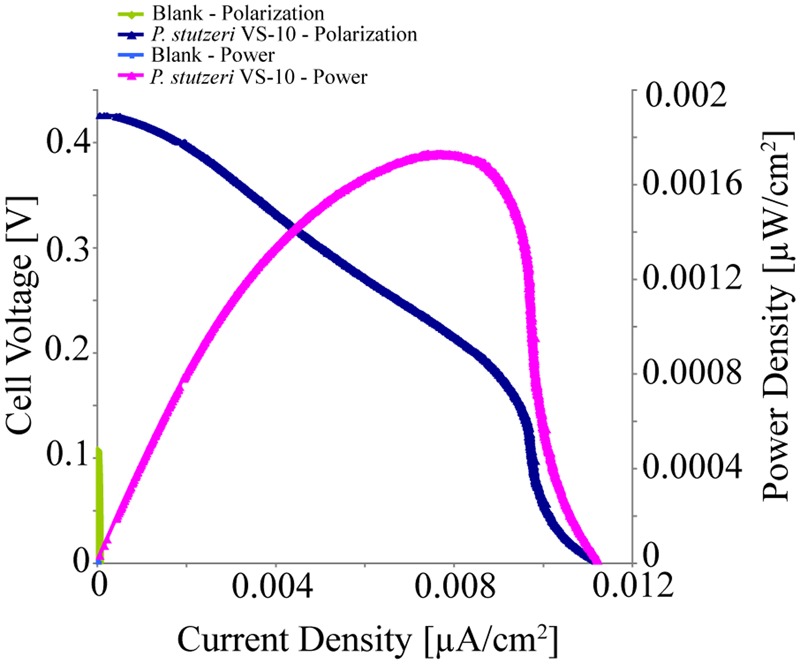
**Polarization and power curves for the microbial fuel cells (MFC) testing of *P. stutzeri* VS-10’s potential to use an anode as a terminal electron acceptor.** The maximum power density of the biotic MFC (pink line) is 1.73 × 10^-3^ μW/cm^2^ compared to an un-inoculated fuel cell (blank) with a maximum power density of 3.07 × 10^-6^ mW/cm^2^ (green line). The maximum current densities for the inoculated and un-inoculated MFCs were 1.12 × 10^-2^ μA/cm^2^ (dark blue line) and 5.90 × 10^-5^ μA/cm^2^ (light blue line, barely visible at the *x, y* origin).

*Pseudomonas stutzeri* VS-10’s ability to use an anode as a terminal electron acceptor and hence transfer electrons to a solid surface was investigated in MFCs under microaerobic conditions over 5 days. It was shown that *P. stutzeri* VS-10 produced an electrochemical current relative to a blank MFC operated under identical conditions (**Figure 4**).

The polarization curve of the MFC inoculated with strain VS-10 showed a maximum power density of 1.73 × 10^-3^ μW/cm^2^ compared to the blank MFC (**Figure 4**). The maximum current densities for the MFC containing *P. stutzeri* VS-10 compared to the blank were 1.12 × 10^-2^ μA/cm^2^ and 5.90 × 10^-5^ μA/cm^2^, respectively. The apparent cell densities (based on optical density measurements at 600 nm) decreased significantly over the 120 h of the experiment (data not shown) most likely due to biofilm formation on the anode (**Figure 3F**), along the membrane and on the glass walls of the MFC.

### Cyclic Voltammetry

Cyclic voltammetry was used to evaluate the redox activity of the anode and the type of extracellular electron transfer mechanisms of *P. stutzeri* VS-10 to the anode. CV is commonly used to study and characterize the electron transfer interactions between microorganisms or microbial biofilms and MFC anodes (Fricke et al., 2008). It is also used to distinguish between direct electron transfer (DET, e.g., via membrane bound cytochromes) and mediated electron transfer (MET, e.g., via conductive filamentous “nanowires” or via excretion of redox active components). CV measurements were performed toward the end of the MFC experiment. Because sweep rates were relatively low (∼25 mV/s) and cells were not depleted of the electron donor, we believe CV conditions to be within the turnover regime. This would indicate that at each potential, all the proteins involved in the pathway were oxidized and reduced multiple times (LaBelle and Bond, 2009).

**FIGURE 5:**
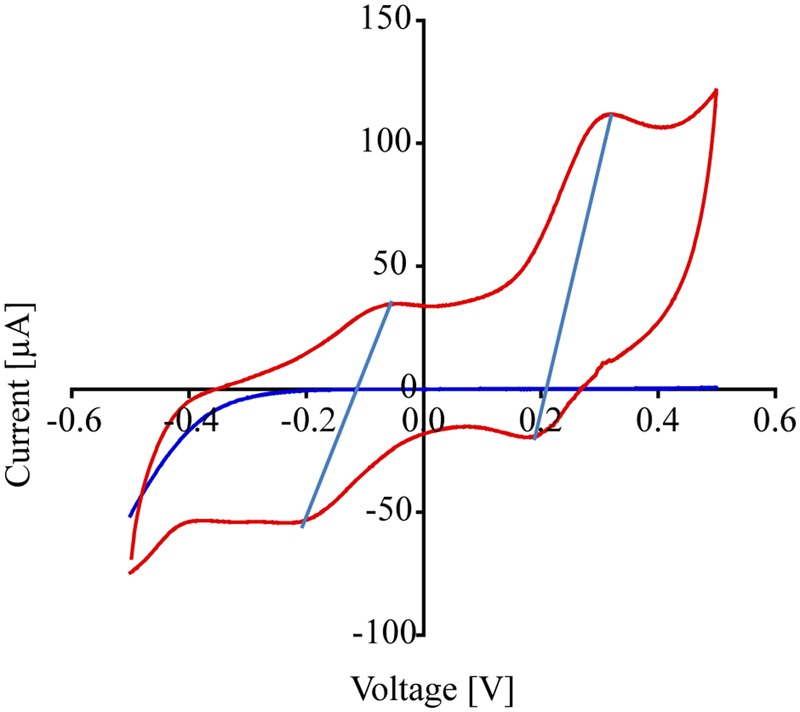
**Results from cyclic voltammogram measurements carried out on the MFC testing for the reduction potential of *P. stutzeri* VS-10.** Cyclic voltammograms of the MFC inoculated with *P. stutzeri* VS-10 (red lines) and an uninoculated control (blank electrode, dark blue line). The presence of at least 2 redox-active components (in form of flat peaks) at ∼ –0.05 V and at ∼0.31 V becomes apparent. The two “peaks” in the upper portion of the current-voltage (I-V) trace are matched by peaks in the lower trace at –0.2 V and +0.18 V. The thin vertical light blue lines connect the peaks in the oxidation and the reduction sweep (a reversible redox reaction).

**Figure 5** shows the cyclic voltammograms of *P. stutzeri* VS-10 (red line) vs. a blank electrode (solid blue line). Data show that the rate of electron transfer increased after inoculation (red line). The voltammogram exhibited two oxidation (anodic) peaks in the upper portion of the current-voltage (I-V) trace that are matched with a light blue line to reduction (cathodic) peaks in the lower trace suggesting that the oxidation and reduction of the corresponding compounds are reversible. The estimated mid-point potentials (E1/2) of the two redox-active compounds are approximately -0.13V and +0.25V (vs. Ag/AgCl) and the difference between the mid-point potential and the anodic and cathodic peak potentials suggests a single electron transfer for both components. It is not known if these peaks represent compounds excreted by VS-10 (e.g., electron shuttles) or if they are directly attached to the cell surface including the filaments.

## Discussion

Weathering of deep ocean basalts has been shown to have a significant impact on local and global ocean chemistry (Hart, 1970; Staudigel et al., 1995, Staudigel et al., 2006). Geological data suggest that seafloor weathering of the oceanic crust is likely to be mediated by microbial activity (Staudigel et al., 2008) but the interactions between submarine basaltic glasses and their associated microbial biofilm communities are, for the most part, unknown. Edwards et al. (2003) were the first to show that basaltic glass, as a source for Fe(II), could support the growth of gamma and alpha proteobacteria characterized as neutrophilic autotrophic FeOB. More recently, Henri et al. (2016) provided the most convincing data from seafloor and laboratory incubations that known lithotrophic FeOB such as the Zetaproteobacterium *Mariprofundus ferrooxydans* PV-1, can utilize structural Fe(II) in basaltic glass as their energy source for growth. While microbial communities associated with submarine basaltic glasses have been shown to be phylogenetically and metabolically diverse, other potential roles of basalt in supporting these communities and the forces that may drive colonization of basaltic glass haven’t been explored in detail.

Basalt surface-related microbial communities show a variety morphological appearances ranging from cell clusters (surface-associated cells) to mature (robust) biofilms (Thorseth et al., 2001; Templeton et al., 2009). These structures have been speculated to correlate with successive stages of colonization starting with incipient microbial colonization by single cells, including many prosthecate organisms (Einen et al., 2006), and developing into more mature microbial communities (Thorseth et al., 1995).

Filamentous structures are often found within microbial biofilms and have more recently become the subject of electrochemical investigations. While microbial extracellular structures have been shown to play a role in electron transfer of microbial strains such as *Shewanella oneidensis* MR-1 and *Geobacter sulfurreducens* (Reguera et al., 2005; El-Naggar et al., 2010; Pirbadian et al., 2014), they were just recently characterized as extensions of the outer membrane in *S. oneidensis* MR-1 (Pirbadian et al., 2014), making them distinct from other extracellular polymeric substances (EPS) commonly excreted by microbes forming the biofilm matrix. The role of such filaments in surface related growth and potentially the electron transport chain of deep-sea bacteria, and heterotrophic FeOB in particular, is, so far, poorly understood.

While substrate associated growth is a dominant microbial lifestyle (i.e., biofilm vs. planktonic communities), it remains ambiguous whether basalt colonization provides an ecological advantage such as acquisition of nutrients and/or energy sources for the surface-associated microbes (Bailey et al., 2009). In addition, the mechanisms of microbial basalt colonization and how surface related bacteria may satisfy their metabolic needs are largely unknown.

To address some of these unknowns we investigated the physiology of an organism we isolated from seafloor volcanic rock, *P. stutzeri* VS-10, a heterotrophic Fe(II)-oxidizing and siderophore-producing bacterium (Sudek et al., 2009). Results from three lines of inquiry in this study provide new insights into the lifestyle of VS-10. First, we showed that *P. stutzeri* VS-10 grows noticeably better without a physical barrier to the basalt while little growth is observed when the organism is separated from the basalt by a permeable membrane that also prevents the diffusion of small molecules; this leads to the recognition that *P. stutzeri* must have mechanisms for acquiring Fe from basalt. Second we described the physical appearance of biofilms on glass surfaces, observing the formation of both filamentous and sheet-like structures, which generated questions regarding the role of such filaments within the biofilm. Finally, we measured some of the electrochemical properties of the surface attached cells, enabling us to hypothesize about their possible role in electron transfer mediated by *P. stutzeri* VS-10.

### Basalt as a Source for Nutritional Fe

Elevated growth under Fe-limited conditions in the presence of basaltic glass (**Figure 1**) suggests that the Fe supplied from the basalt is used by cells either as a nutrient and/or energy source. Based on diffusion chamber experiments, either direct attachment or at least physical proximity of cells to the glass surface is critical in this process (**Figure 2**).

*Pseudomonas stutzeri* VS-10 was originally isolated as a siderophore-producing bacterium. It was shown to produce a number of structurally related siderophores (amonabactins) when grown under Fe-limited conditions on a minimal glycerol medium (Sandy, 2011; Sudek, 2011). Basaltic glasses used in our study have the potential to provide nutritional Fe in two forms, directly as structurally-bound Fe(II) and Fe(III), or in the form of Fe(hydr)oxides formed in the presence of oxygen along the basalt surface. These Fe(hydr)oxides are commonly found on natural submarine basalt surfaces and are believed to be the result of both biotic and abiotic oxidation of the basalts.

Perez et al. (2016) recently demonstrated the essential role of siderophores in the *P. aeruginosa*-promoted dissolution processes of basaltic glasses. This work revealed that the Fe speciation in the glass could affect siderophore production, and in turn, *P. aeruginosa* could affect the basalt dissolution rates and take up released Fe as a required nutrient for growth. The role of siderophores produced by *P. stutzeri* VS-10 in providing nutritional Fe and subsequently supporting growth in our experiment is unknown. Results from diffusion chamber experiments (**Figure 2**) may indicate an involvement represented by a lower growth under conditions where siderophore diffusion through a membrane and subsequent complexation of Fe from basalt or Fe(hydr)oxides was prevented. If siderophores were indeed involved in nutritional Fe acquisition from basalt that would most likely affect basalt dissolution rates over time as described for *P. aeruginosa* (Perez et al., 2016).

### Basalt as an Electron Source or Sink

Electrochemical data indicate that *P. stutzeri* VS-10 cells appear to be able to use solid surfaces both as an electron donor and a terminal electron acceptor (**Table 2** and **Figure 4**). Such properties could enable this strain to either use the basalt as an electron source, facilitating the oxidation of structural Fe(II), or to the use of Fe(III) oxides formed on the basalt surface as an electron sink. Both scenarios would benefit the growth of the bacterium energetically. Such physiological interactions are deserving of further study to determine the potential mechanisms of electron transfer from solid-phase Fe(II) and the resulting impact on the chemical properties of the basalt/microbe interface.

The role of filaments observed in the biofilm structure, and/or redox-active compounds such as electron shuttles in the establishment of a biofilm and in elevated growth of *P. stutzeri* VS-10 on the basalt surface is intriguing. We hypothesize that either one could be involved in oxidizing Fe(II) from basalt or reducing surface associated Fe(hydr)oxides. This would again be supported by our diffusion chamber experiments in which a direct attachment to the rock (filaments) or the diffusion of redox-active compounds through the membrane was prevented.

While soluble components, possibly redox shuttles or redox mediators as suggested by cyclic voltammograms of *P. stutzeri* VS-10 (**Figure 5**), could be used as part of the strain’s electron transfer pathway, the exact structure of such putative compounds is unknown. Possible electron shuttles include organic heterocyclic and monocyclic compounds, sulfur species and hydrogen (Rabaey et al., 2007). Many of these chemical species have to be provided externally for the bacterium to be able to use them for shuttle-based electron transfer. However, *Pseudomonas* species commonly produce exogenous redox-active compounds such as the phenazines, pyocyanin and phenazine-1-carboxamide (Hernandez et al., 2004; Rabaey et al., 2005; Yong et al., 2014). Bacterial phenazines are commonly known to serve as electron shuttles to alternate terminal acceptors, to modify cellular redox states and to contribute to biofilm formation and architecture (Pierson and Pierson, 2010).

While the abundance of filamentous structures in bacterial biofilms from variable environmental habitats seems to be far more common than initially thought, the role of such filaments in metabolic processes remains for the most part unknown. The research presented here represents a first hypothesis on their role in supporting life on submarine basaltic glasses.

The lack of experimental replicates does not allow robust comparisons of our electrochemical results to previously published data on *S. oneidensis* MR-1, an electrogenic bacterium that has been studied in detail. However, comparisons between our polarization and maximum current density results for *P. stutzeri* VS-10 and *S. oneidensis* MR-1 were attempted to be made (**Table 2**). Overall these results suggest that *P. stutzeri* VS-10 is able to build a larger cathodic potential than *S. oneidensis* MR-1 (i.e., it has a higher oxidation potential than MR-1) while *S. oneidensis* MR-1 seems to be more efficient at stripping electrons from the cathode. The electrochemical similarities between *P. stutzeri* VS-10 and *S. oneidensis* MR-1 biofilms may indicate that the electron transport system of *P. stutzeri* VS-10 is expressed on the cell surface and that there are distinct electron pathways into and out of the cells as previously described in *S. oneidensis* MR-1 (Bretschger et al., 2007).

One phenomenon we observed in *P. stutzeri* VS-10 cultures containing basalt was the occurrence of what seems to be secondary mineral deposits (most likely metal oxides) along the filamentous structures. The encrustation is extensive and seems to correlate with biofilm thickness and/or length of growth. Single cells or monolayers often still appear clean after a few days (**Figures 3B,C**) in comparison to thicker parts of the biofilm that established over longer periods of times. In biofilms that had more time to develop, encrustation of filaments is intense while cells still appear comparatively clean (**Figures 3A,D**). Indication that these precipitates represent metal oxides derived from the alteration of basalt is the fact that they are lacking in cultures cultivated inside MFCs where electrons are provided from a cathode or attached to an anode (**Figure 3F**).

Precipitation of Fe(III)-oxides (generated as waste products) on the cell surface and the resulting encrustation of cells, potentially impeding cell metabolism, represent a common problem for neutrophilic Fe(II)-oxidizing bacteria. The metal oxides may give rise to limited diffusion of nutrients and restricted transport of waste across the membrane resulting in a reduction in overall viability of the cell (Kappler et al., 2005). Potential strategies to avoid such encrustation and to ensure mineral deposition at a certain distance from the cell surface have been reviewed previously (Schaedler et al., 2009). The most common mechanism described is the precipitation of Fe(III)-minerals along extracellular organic matrixes (sheaths, stalks, filaments or fibers) which are considered precipitation templates or nuclei. Such precipitation has been described in several species of neutrophilic Fe(II)-oxidizing bacteria including *Rhodobacter ferrooxidans* strain SW2, *Gallionella* spp., *Chlorobium ferrooxidans* strain KoFox, *Leptothrix ochracea* and more recently *Mariprofundus ferrooxidans* PV-1 (Chan et al., 2004; Hallberg and Ferris, 2004; Summers et al., 2013).

*Pseudomonas stutzeri* VS-10’s ability to accelerate Fe(II)-oxidation under heterotrophic conditions could represent a metabolic trait to ensure additional energy acquisition in oligotrophic environments. *P. stutzeri* VS-10 biofilm growth on basaltic glass has been shown to be substantial. Diffusion limitation within the biofilm as commonly described Stewart (2003) is likely. We speculate that basalt may serve as an additional energy source in *P. stutzeri* VS-10 biofilms. In particular, cells in direct contact with basalt are more likely to become subject to diffusion limitation. Biologically catalyzed Fe(II)-oxidation at this interface could therefore represent a metabolism that is thermodynamically and kinetically favorable for microbial utilization at the lower O_2_ saturation within a biofilm. This correlates well with the enhanced growth characteristics of strain VS-10 when grown in close contact with basaltic rock and the ability to grow on the cathode in a MFC.

Summers et al. (2013) recently demonstrated that cathodes poised at a constant redox potential, designed to mimic the one of Fe(II) in the environment, could serve as an electron donor to the marine Fe(II)-oxidizing bacterium *Mariprofundus ferrooxydans* PV-1. In comparison to *P. stutzeri* VS-10, PV-1 cells were shown to be non-biofilm forming and appeared to attach to the cathode via single cell polysaccharide matrixes. However one phenomenon both strains have in common is the presence of Fe(III)-minerals along extracellular polymers (stalks in PV-1; filaments in VS-10) in cultures containing Fe and the absence of such in MFCs.

Based on our results we hypothesize that extracellular filaments, most likely representing an extension of the cells outer membrane and possibly being involved in the electron transport chain of bacteria, may be more common in marine heterotrophic Fe(II)-oxidizing microorganisms than currently appreciated. The potential role in electron-transfer reactions between rock surfaces and microbial biofilms may be critical for promoting important redox processes necessary to sustain a diverse deep-ocean biosphere.

Recently, Rowe et al. (2015) adopted a strategy to used poised electrodes as a cultivation approach to enrich for electrochemically active organisms from marine sediments, and to subsequently select for organisms that could use solid substrates as an electron donor. In that study, Fe(0) and FeS were used as the electron donors to isolate Fe-oxidizers from the genera *Halomonas, Marinobacter, Pseudomonas*, and *Idiomarina* from the electrode enrichments, which were then subjected to electrochemical tests. All of these isolates were shown to be heterotrophic as well as FeOB, and current production would occur through electrode oxidation in the presence of O_2_. This study did not provide direct evidence for redox mediators, and attachment to the electrode was common. This work serves as another example where *Pseudomonas* strains have been shown to be metabolically versatile and capable of growing using an electrode as the electron donor.

We previously suggested that metabolic versatility in microorganisms is likely to represent a common trait in oligotrophic deep-sea environments (Sudek et al., 2009). Siderophore production is a mechanism developed to ensure nutritional Fe-availability in deep-sea waters where Fe solubility is commonly low. In contrast, the ability to harvest energy through the oxidation of both Fe(II) and organics could represent an adaptive mechanism enabling a mixotrophic lifestyle and providing the option to thrive under geochemically dynamic conditions. Similarly, the ability to use the anode as an electron acceptor suggests that oxidized metal species commonly found on basalt surfaces could represent accessible electron acceptors for microbes growing on surfaces in diffusion limited biofilms. The results presented here underscore the potential role of submarine basaltic glass to act both as a source for nutrients and for energy generation subsequently supporting the phylogenetically and metabolically versatile microbial communities within deep-sea ecosystems.

## Author Contributions

LS designed and performed all the experiments and interpreted the results. GW helped to design, run and interpret the electrochemical analyses (microbial fuel cells, conductivity measurements). LS wrote the manuscript. BT, AT, and HS substantially contributed to the experimental design, data interpretation, drafting and revising of the manuscript. LS, GW, AT, HS, and BT all agree to be accountable for all aspects of this work and approved the final version to be published.

## Conflict of Interest Statement

The authors declare that the research was conducted in the absence of any commercial or financial relationships that could be construed as a potential conflict of interest. The reviewer AR declared a shared affiliation, though no other collaboration, with one of the authors GW to the handling Editor, who ensured that the process nevertheless met the standards of a fair and objective review.
